# MoSe_2_ Nanoflowers for Highly Efficient Industrial Wastewater Treatment with Zero Discharge

**DOI:** 10.1002/advs.202102857

**Published:** 2021-10-24

**Authors:** Jyun‐Ting Lee, Shaurya Mathur, Sophia Shen, Jyh‐Ming Wu, Jun Chen

**Affiliations:** ^1^ Department of Materials Science and Engineering National Tsing Hua University Hsinchu 300 Taiwan; ^2^ Department of Bioengineering University of California, Los Angeles Los Angeles CA 90095 USA; ^3^ High Entropy Materials Center National Tsing Hua University Hsinchu 300 Taiwan

**Keywords:** eco‐friendly, MoSe_2_ nanoflowers, ultrahigh concentration, wastewater treatment, zero discharge

## Abstract

Water pollution is one of the leading causes of death and disease worldwide, yet mitigating it remains a challenge. This paper presents an efficient new strategy for the processing of wastewater utilizing an accessible redox reaction with MoSe_2_ nanoflowers, which shows a strong oxidizing ability and permits the decomposition of dye molecules in dark environments without the need for an external power source. This reaction can treat wastewater at a decomposition rate above 0.077 min^−1^, even when interacting with organic pollutants at concentrations up to 1500 ppm. Theoretical calculations by Dmol^3^ simulation elucidates that the reactions proceed spontaneously, and the kinetic constant (*k*_obs_) for this redox reaction with 10 ppm RhB dye is 0.53 min^−1^, which is 65 times faster than the titanium dioxide photocatalytic wastewater treatment. More importantly, the residual waste solution can be further utilized as a precursor to reconstruct the MoSe_2_ nanoflowers. To demonstrate the effectiveness and reusability, the treated effluent is directly used as the sole source of irrigated water for plants with no adverse effect. This method offers an eco‐friendly and more accessible way to treat industrial wastewater with zero‐discharge.

## Graphical abstract

In this work, an efficient new strategy is presented for the processing of wastewater utilizing an accessible redox reaction with MoSe_2_ nanoflowers, which shows a strong oxidizing ability and permits the decomposition of dye molecules in dark environments without the need of an external power source.

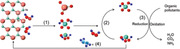

## 1 Introduction

The developmental level of a country is directly linked its water accessibility, as it affects not only the health of their citizens but also the health of their ecosystems. In fact, water pollution is one of the leading causes of death and disease worldwide^[^ ^1^, ^2^ ^]^ and can cause damage to natural flora and fauna and their overarching biological ecosystems.^[^ ^3^, ^4^, ^5^ ^]^ Water can be polluted in a wide variety of ways from many different pollutants, but one of the most impactful is wastewater produced by industrial contamination, which contains a wide range of different pollutants at different concentrations and combinations. To clean the water these pollutants need to be processed at a high level of safety and effectiveness, but also at a relatively low cost in order to maintain accessibility to the public. This can be done by using methods that do not require a high amount of technical knowledge or labor to perform, which can reduce labor costs.^[^ ^6^ ^]^ Currently, the price of wastewater processing is extremely high, and is projected to reach nearly 456 billion dollars worldwide in 2026 due to population growth and freshwater shortages.^[^ ^7^ ^]^ Thus, developing an efficient, low‐cost, and scalable technology to process wastewater is an urgent issue to tackle.

Current wastewater treatment methods include a multitude of physical, chemical, and biological processes including solvent extraction,^[^ ^8^ ^]^ precipitation,^[^ ^9^ ^]^ carbon adsorption,^[^ ^4^ ^]^ flotation,^[^ ^10^, ^11^ ^]^ ion exchange,^[^ ^12^, ^13^, ^14^ ^]^ membrane filtration,^[^ ^15^, ^16^ ^]^ electrochemical methods,^[^ ^17^, ^18^ ^]^ bioremediation,^[^ ^19^ ^]^ and advanced oxidation process technologies.^[^ ^20^, ^21^, ^22^, ^23^, ^24^ ^]^ Conventional chemical and physical water treatment are economically advantageous, technologically simple, efficient, and offer a wide range of commercial applications. However, the high construction and repair‐maintenance costs, high energy consumption, and sludge production are major disadvantages to using these processes to treat polluted water.^[^ ^25^, ^26^ ^]^ Biological processes involve the use of microbes and are thus eco‐friendly, cost‐effective, and have diverse amount of processing options, but maintaining a well‐controlled environment for the microbe is difficult limiting microbial degradation efficiency.^[^ ^27^ ^]^ As a result, many alternate methodologies have been developed to increase the efficiency of the treatment process such as a fenton‐like system,^[^ ^28^ ^]^ electro‐fenton,^[^ ^29^, ^30^, ^31^ ^]^ photocatalytic processes,^[^ ^32^, ^33^ ^]^ and piezocatalysis.^[^ ^34^, ^35^, ^36^, ^37^ ^]^ However, other issues such as sludge production,^[^ ^21^ ^]^ electrolyte addition, short‐lived reactive species,^[^ ^33^, ^38^ ^]^ and catalyst deactivation^[^ ^39^ ^]^ limit their commercialization. The limitations with current treatment methods highlight the need to create a sustainable and accessible wastewater treatment technology that can treat large amounts of water with ultrahigh concentrations of pollutants by integrating innovative resource recovery technologies into treatment‐process designs.^[^ ^40^ ^]^

Herein, we developed a new degradation method using molybdenum diselenide nanoflowers (MoSe_2_ NFs) and hydrogen peroxide (H_2_O_2_) solution to form an oxidized solution. This oxidized solution exhibits excellent degradation activity and can treat ultra‐high concentrations (1500 ppm) of Rhodamine B (RhB) organic pollutants, a commonly used dye in industrial processes.^[^ ^41^, ^42^, ^43^ ^]^ Moreover, this method can lead to 100% wastewater reusability, as the treated effluent can be used as a watering source for plants with no notable negative outcomes on the plants’ growth. Furthermore, the treated effluent can be employed as a precursor to re‐synthesize MoSe_2_ NFs by adding hydrazine through the hydrothermal process, achieving the aim of zero discharge in a wastewater treatment process. Calculation of all Gibbs free energy change (∆*G*) reactions are shown in this work, and the results demonstrate the entire degradation process is a spontaneous chain reaction that can be performed in dark conditions without light irradiation.

## 2 Results and Discussion

### 2.1 Working Principle and Material Characterization

**Figure 1:**
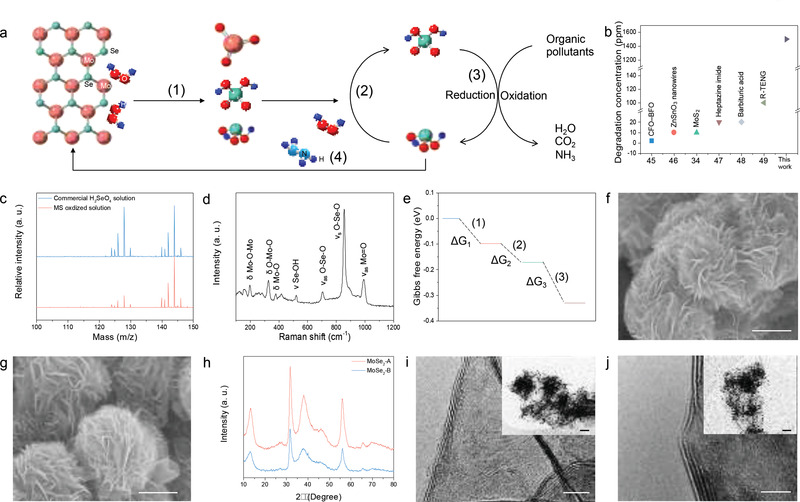
a) The schematic diagram of zero‐discharge system. b) The comparison of concentration for dye removal for the work and the recent reports in premier journals. c) ESI‐MS spectrum of commercial H_2_SeO_4_ and MS oxidized solution. d) The Raman spectrum of MS oxidized solution. e) The reaction Gibbs free energy change of Equations ((1), (2), (3)). f) The SEM image shows the petal‐like morphology of MoSe_2_‐A NFs. Scale bar: 500 nm. g) The SEM image of MoSe_2_‐B NFs. Re‐synthesized MoSe_2_‐B NFs have the same surface morphology as MoSe_2_‐A NFs. Scale bar: 500 nm. h) The XRD pattern of MoSe_2_‐A and ‐B, respectively. i) The TEM images of MoSe_2_‐A NFs show that the interlayer spacing is 0.61 nm. Scale bar: 5 nm. Inset, low magnification TEM image of MoSe_2_‐A NFs. Scale bar: 100 nm. j) The TEM images of MoSe_2_‐B NFs show that the interlayer spacing is 0.62 nm, consistent with MoSe_2_‐A NFs. Scale bar: 5 nm. Inset, low magnification TEM image of MoSe_2_‐B NFs. Scale bar: 100 nm.

The decomposition of dye molecules in the zero‐discharge system can be described in four steps, as illustrated in **Figure** **1**a: 1) The electrolyzed H_2_O_2_ mixes with MoSe_2_ NFs. 2) The solution, which now has the potential to oxidize wastewater, forms a thermodynamic nonequilibrium state of H_2_SeO_4_ and H_2_SeO_3_ (the MS oxidized solution). 3) The ultrahigh concentration of dye molecules (>1500 ppm) is efficiently decomposed by the MS oxidized solution. At the same time, H_2_SeO_4_ is reduced to H_2_SeO_3_, which is further oxidized by H_2_O_2_ back into H_2_SeO_4_, allowing for continuous decomposition of organic components and demonstrating the ultrahigh degradation capabilities of the MS oxidized solution. 4) The MoSe_2_ NFs are re‐synthesized through the hydrothermal process by adding hydrazine into the treated solution. A comparison of the dye removal concentrations between our zero‐discharge system and many recent reports in premier publications is shown in Figure 1b, demonstrating its high degradation concentration. In fact, our process can decompose the highest dye concentration ever reported (Table S1, Supporting Information).^[^ ^34^, ^45^, ^46^, ^47^, ^48^, ^49^, ^50^, ^51^, ^52^ ^]^

To further understand the degradation mechanism, negative ion mode electrospray ionization‐ion trap mass spectrometry (ESI‐MS) was used to investigate the deprotonated molecules in the MS oxidized solution, using a control sample prepared with a commercial selenic acid (H_2_SeO_4_) solution (40 wt. % in H_2_O, Sigma‐Aldrich) (Figure 1c). In the ESI‐MS spectra, the molecular ion region lines were located at *m*/*z* values of 127.9 and 143.9 which were attributed to HSeO_3_^+^ and HSeO_4_^+^, indicating that the commercial selenic acid contained both compounds (upper spectrum). In addition, the intensity in the ESI‐MS spectra of both H_2_SeO_3_ and H_2_SeO_4_ were identical, indicating a state of thermodynamic equilibrium in the solution. In comparison, the MS oxidized solution contained the same molecular ion region lines as selenic acid, but the intensity of H_2_SeO_4_ was significantly higher than H_2_SeO_3_, indicating an extremely non‐equilibrium thermodynamic state (lower spectrum). The dramatically increased formation of H_2_SeO_4_ in MS oxidized solution is further demonstrated by a comparison of the ESI‐MS spectra of the commercial selenic acid solution and MoSe_2_ NFs mixed with various concentrations of H_2_O_2_ from 0 to 90% (Figure S1, Supporting Information). Here, when the H_2_O_2_ concentration increased, this created H_2_SeO_3_ with by‐products of MoO_3_, H_2_, and O_2_, as expressed by Equation (1).

(1) $$2\mathrm{MoS}e_{2}+11H_{2}O_{2\;}\to \;4H_{2}\mathrm{Se}O_{3}+2\mathrm{Mo}O_{3}+7H_{2}+2O_{2}$$

The H_2_SeO_3_ in the presence of high concentrations of H_2_O_2_ reacted to form H_2_SeO_4_, enhancing the concentration of H_2_SeO_4_ and forming the MS oxidized solution with H_2_O, as shown in Equation (2).

(2) $$H_{2}\mathrm{Se}O_{3}+H_{2}O_{2}\;\to H_{2}\mathrm{Se}O_{4}+H_{2}O$$

Consequently, the MS oxidized solution, which contains a high concentration of H_2_SeO_4_ solution, decomposes the dye molecules (i.e., RhB, C_28_H_31_ClN_2_O_3_) to form H_2_SeO_3_ with by‐products of CO_2_, NH_3_, Cl_2_, and H_2_O, as given by Equation (3).

(3) $$\begin{array}{ccc} & & 131H_{2}\mathrm{Se}O_{4}+2C_{28}H_{31}\mathrm{Cl}N_{2}O_{3}\to 131H_{2}\mathrm{Se}O_{3}+56CO_{2}\\ & & \;+4NH_{3}+Cl_{2}+25H_{2}O \end{array}$$

Subsequently, a chain reaction occurs, allowing the H_2_SeO_3_ to further oxidize the dye molecules by transferring them into the H_2_SeO_4_.

Raman spectroscopy of the MS oxidized solution exhibited modes characteristic of MoO_3_, H_2_SeO_3_, and H_2_SeO_4_ in the range of 100–1200 cm^−1^ (Figure 1d). The characteristic peaks at 196 and 326 cm^−1^ represent the bending vibration of Mo—O—Mo and O—Mo—O bonds, respectively, while the 377 cm^−1^ peak is associated with scissoring vibration modes of O—Mo—O.^[^ ^53^, ^54^ ^]^ Additionally, the peak situated at 991 cm^−1^ is assigned to the asymmetric stretching vibration of the Mo═O bond, while the 520 cm^−1^ peak is attributed to the stretching vibration of the Se—OH bond. Finally, the 704 and 856 cm^−1^ peaks correspond to the asymmetric and symmetric stretching vibration of the O—Se—O bond, respectively.^[^ ^55^, ^56^ ^]^ Next, using the first‐principles simulation in the DMol^3^ module in Materials Studio, the ∆G was calculated, with Δ*G*_1_, Δ*G*_2_, and Δ*G*_3_ as −0.098, −0.073, and −0.157 eV, respectively, which implies that the degradation processes are spontaneous without any provided additional energy at 298.15 K and 1 atm. (Figure 1e). Detailed calculation steps can be found in Note S1, Supporting Information. Figure 1f reveals the SEM image of the MoSe_2_ NFs (MoSe_2_‐A), which exhibit abundantly flower‐like morphologies with 3D hierarchical architecture nanosheets highly dispersed around the edge sites. After decomposing the dye molecules, the treated solution was further used as a precursor for preparing the MoSe_2_ NFs precursor by adding additional hydrazine (MoSe_2_‐B), whose reconstruction process is shown in Figure S2, Supporting Information. First, the 2 mL hydrazine was used as the reducing agent to reduce H_2_SeO_4_, H_2_SeO_3_, and MoO_3_ to Se and Mo monomers, which adjusted the pH value to 7. Next, the solution was transferred to an autoclave for the hydrothermal process (220 °C for 24 h). Finally, after centrifugation and drying, the reconstructed MoSe2‐B can be obtained. The morphologies of MoSe_2_‐B are very similar to MoSe_2_‐A, as revealed through the SEM image of MoSe_2_‐B (Figure 1g). The XRD diffraction patterns have also confirmed that both MoSe_2_‐A and ‐B exhibited the same P6_3_/mmc space group (JCPDS Card No. 87‐2419), as shown in Figure 1h. Compared to the MoSe_2_‐A, the MoSe_2_‐B was slightly shifted toward the low‐angle region, suggesting that residual strain was created in MoSe_2_‐B. However, both MoSe_2_‐A and ‐B show a significant exposure of active edge sites, which provide a high specific surface area to facilitate rapid reaction with H_2_O_2_ (Figure 1i,j). Additionally, they show the same geometry, morphology, crystal structure, and compositions (see Figure S3, Supporting Information). Our work reveals that there is no residual waste solution discharge to the environment and in addition, the whole degradation process proceeded with dark conditions, achieving a zero‐discharge system.

### 2.2 Evaluation of Degradation Activity

**Figure 2:**
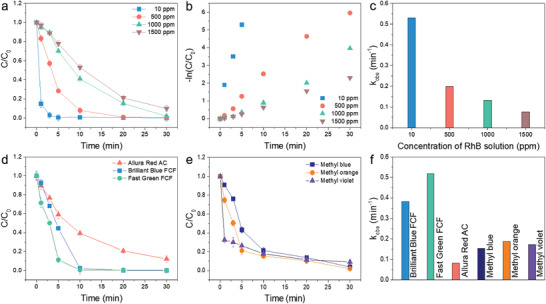
a) The degradation activity of RhB solution by MS oxidized solution at different degradation time intervals. Reaction conditions are 10 mg MoSe_2_ NFs, 10 mL 90% H_2_O_2_ solution, 10 mL RhB solution. b) The first‐order kinetic plot and c) degradation rate constant of various concentrations RhB solution. The degradation curve of d) food colorings (i.e., allura red ac, brilliant Blue FCF, and fast green FCF) and e) industrial dyes (i.e., methyl‐blue, ‐orange, ‐violet) at different degradation time intervals and the f) corresponding degradation rate constant. Reaction conditions are 10 mg MoSe_2_NFs, 10 mL 90% H_2_O_2_ solution, 10 mL food colorings, and dye solution, respectively.

The efficiency of the system for wastewater treatment can be evaluated by its degradation activity. As shown in **Figure** **2**a, the dye removal rate is inversely proportional to the dye concentration. This result reveals that after 30 min, the decolorization efficiency reached 100% for dye concentration in the range of 10 to 1000 ppm. Furthermore, even at dye concentrations as high as 1500 ppm, the removal ratio still reached 80%. The dye degradation kinetic constant can be calculated using $\;k_{\mathrm{obs}}=\;\frac{-\ln(\frac{C}{C_{0}})}{t}$, where *k*_obs_ (min^−1^) is the pseudo‐first‐order rate constant for decolorization of the dye solution by the MS oxidized solution and yields a straight line with relatively high regression coefficients (*R* ^2^ > 0.95), as shown in Figure 2b. Calculation of the kinetic reaction constant (*k*_obs_) for different dye concentrations of 10, 500, 1000, 1500 ppm yielded values of 0.53, 0.19, 0.14, 0.077 min^−1^, respectively (Figure 2c). The highest *k*_obs_ constant reached 0.53 min^−1^ for the 10 ppm dye molecules, which is 65 times faster than conventional titanium dioxide (TiO_2_) photocatalysis.^[^ ^44^ ^]^ Moreover, the *k*_obs_ using the MS oxidized solution with 1500 ppm dye solution was 0.077 min^−1^, which is still superior to using the conventional photocatalytic process for a 10 ppm dye solution. Figure S4, Supporting Information, shows the positive ion ESI mass spectra of the 1500 ppm RhB degradation process, and the lack of other intermediates implies that RhB is directly decomposed. This is further demonstrated by Figure S5, Supporting Information, which shows no degradation activity of the control sample (pristine 90% H_2_O_2_). These results support the conclusion that the dye degradation ability results from the MS oxidized solution, rather than the high concentration H_2_O_2_solution. The versatility of our process is further demonstrated in Figure 2d,e, where we used the MS oxidized solution to decompose high concentration (1500 ppm) of food colorings (i.e, Allura red ac, brilliant blue FCF, and fast green FCF) and industrial dyes (methyl‐ blue, ‐orange, ‐violet). The *k*_obs_ in 30 min for the different food colorings and industrial molecules can be found in Figure 2f. These results demonstrate that this process could be widely used for the decomposition of various dye molecules with a high‐efficiency decomposition rate (> 0.1 min^−1^).

### 2.3 Exploring of Industrialization

**Figure 3:**
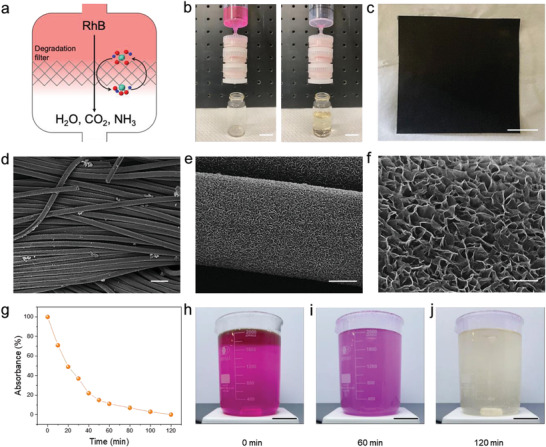
a) Concept sketch of degradation filter. b) Photos of the Video S2, Supporting Information. Scale bar: 5 cm. c) Digital photo of a large area (225 cm^2^) carbon felt with grown the MoSe_2_ NFs on the surface. Scale bar: 5 cm. d) The SEM image of the carbon felt showing that there are many voids between the carbon fibers. These voids provide channels for flowing of pollutants and allow them to react with MoSe_2_ NFs. Scale bar: 50 µm. e) The SEM image shows that the MoSe_2_ NFs have grown on the carbon fiber uniformly. Scale bar: 5 µm. f) The SEM image shows the petal‐like MoSe_2_ NFs morphology that provides a lot of reaction sites for the degradation process. Scale bar: 1 µm. g) Degradation of large volume RhB solution. The reaction conditions are 1 g MoSe_2_ NFs, 1000 mL RhB (10 ppm), and 1000 mL 90% H_2_O_2_. Digital photo of the degradation video #2 at h) initial state, i) 60  min, and j) 120 min. The RhB molecules are gradually decomposed. Scale bar: 8 cm.

To simulate the decomposition of the organic pollutants systemically, a degradation filter was fabricated using a syringe, as shown in the schematic diagram of **Figure** **3**a. The degradation ability of the filter was examined by passing a mixed solution of 10 ppm RhB and 90% H_2_O_2_ through the syringe filter with a constant flow rate (2 mL min^−1^) controlled by a linear motor. As shown in Figure 3b, the effluent colors indicated that the MoSe_2_ syringe filter maintained a 100% dye removal ratio. The complete degradation of life video is shown in Video S1, Supporting Information. To create this highly efficient filter, a syringe filter was first decorated with MoSe_2_ NFs by filling it with carbon felt and then the MoSe_2_ NFs were uniformly grown on its surface, which has an area of 1.8 cm^2^ (Figure 3c). The SEM images, shown in Figure 3d–f, illustrate the high specific surface area of MoSe_2_ NFs uniformly grown on the surface of carbon felt. Additionally, the dye removal rate of large volume of RhB dye solution (concentration ≈10 ppm), which is necessary to evaluate for industrial applications, reached a steady state of almost zero after 120 min as illustrated by the degradation curve in Figure 3g. Live Video S2, Supporting Information, further demonstrates the full decomposition of the RhB dye by the MS oxidized solution within 120 min. Corresponding photographs of this decomposition process are shown for 0, 60, and 120 min in Figure 3h–j. These results indicate that the MS oxidized solution is highly promising for industrial application.

### 2.4 The Toxicity Test of the Treated Water by Plants Assay

**Figure 4:**
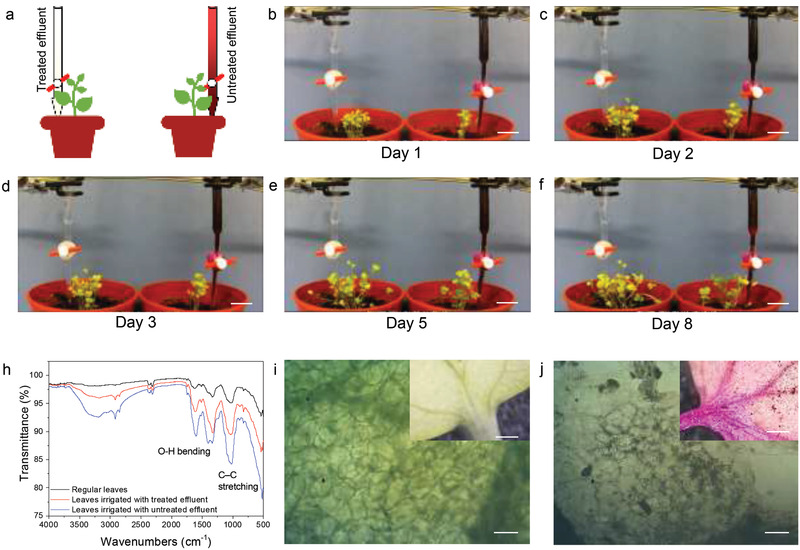
a) Schematic drawing of plant growing experiment setup. The treated and untreated effluent are filled in two burettes and the cabbage seedlings are irrigated with the same flow rate, respectively. The photo showing the plant growing video (b–f) at day 1 to day 8. The cabbage seedlings irrigated with treated effluent have grown healthy. In contrast, the cabbage seedlings irrigated with untreated effluent are dyed, shriveled, and withered. Scale bar: 3 cm. d) The FTIR spectrum of regular leaves and the leaves irrigated with treated effluent and untreated effluent, respectively. e) The OM images of the leaf samples irrigated with treated effluent. Healthy mesophyll cells are clearly observed. Scale bar: 5 µm. Inset, low magnification image of the leaf. Scale bar: 1  mm. f) The OM images of the leaf samples irrigated with untreated effluent. The mesophyll cells have shrunk and been destroyed. Scale bar: 5 µm. Inset, low magnification image of the leaf. Scale bar: 1 mm.

To achieve 100% reuse, the treated effluent could be used to grow plants. Here, cabbage seedlings were continuously irrigated with either the treated or untreated effluent to observe the growth of the seedlings, as shown in the schematic diagram in **Figure** **4**a. The seedlings were grown for 8 days, whose progress is shown via images in Figure 4b–f, while Video S3, Supporting Information, contains the time‐lapse video of the seedlings growing. The untreated RhB solution caused acute toxicity with many adverse effects on cabbage seedling growth. In contrast, the cabbage seedling which was irrigated with the treated effluent sustained healthy growth. As shown in Figure 4h, Fourier‐transform infrared spectroscopy (FTIR) spectra of leaves irrigated with untreated effluent contained pronounced C—H and C—O bond peaks caused by atrophic mesophyll cells, which also resulted in additional bands for O—H stretching at 1400 cm^−1^ and C—C bonding at 1238 cm^−1^. The leaf mesophyll cells are the main contributors to photosynthetic activity, so the contamination of the mesophyll cells strongly affected the plant's growth. In contrast, the spectrum of leaves irrigated with treated effluent showed no difference to regular leaves. Furthermore, as shown in their optical microscope (OM) images and inset images in Figure 4i–j, the cabbage that was irrigated by the untreated effluent resulted in, destroyed and atrophied mesophyll cells. However, when irrigated with the treated effluent, the mesophyll cells were able to grow and maintained the mesophyll cell structure of a regular cabbage (Figure S6, Supporting Information). These results are evidence that of the viability of reusing treated wastewater for crop irrigation. This work demonstrates an up‐and‐coming method for treating the diversification of organic water pollutants using MS oxidized solution to decompose various colors of industrial dye and food coloring molecules with the fastest reported decomposition rate and achievement of zero discharge, high reliability, and 100% reusable water, which could reduce the environmental damage of discharged effluent.

## 3 Conclusion

The simple process of processing wastewater with MS oxidized solution resulted in a high‐efficiency, scalable, and cyclic process for treating ultra‐high concentrations of diversifying organic pollutants. The MoSe_2_ NFs were added with H_2_O_2_ solution to generate a solution that contains a thermodynamic nonequilibrium state of H_2_SeO_4_ and H_2_SeO_3_, which possesses a strongly oxidizing ability for the decomposition of ultrahigh concentrations of up to 1500 ppm various dye molecules, including both industrial dyes and food coloring, with a *k*_obs_ of 0.077 min^−1^. After decomposing the dye solution, the treated effluent can be used as a source of water for plant irrigation without any adverse effects on the plants. Alternatively, the treated effluent containing H_2_SeO_4_ and H_2_SeO_3_ can be used as a precursor for the reconstruction of the MoSe_2_ NFs by adding hydrazine, which achieves an entirely zero discharge system by reusing 100% of the wastewater by‐products. The fastest noted reaction constant (*k*_obs_) is 0.53 min^−1^, acting on a solution with a dye concentration of 10 ppm, which is 65 times faster than titanium dioxide photocatalysis. The entirely spontaneous degradation process occurs in dark conditions without external energy, which is a significant breakthrough for ultra‐high concentration wastewater treatment and a promising method for industrial applications.

## 4 Experimental Section

### MoSe_2_ NFs Synthesis Method

The MoSe_2_ NFs were prepared using the hydrothermal method.^[^ ^57^ ^]^ 0.206 g of Na_2_MoO_4_·H_2_O and the 0.222 g of SeO_2_ powder were dissolved in 58 mL of deionized water. The solution was stirred for 20 min to disperse completely, followed by the slow addition of 2 mL of hydrazine into the solution. After that, the solution was transferred into a teflon‐lined stainless‐steel autoclave and maintained at 220 °C for 24 h. After the heating process, the autoclave was cooled naturally down to room temperature to obtain the MoSe_2_ NFs.

### Characterization Analysis

An X‐ray diffractometer (Bruker D2) with monochromatic Cu K*α* radiation and a cold‐field‐emission scanning electron microscope (Hitachi SU‐8010) were used to characterize the crystal phase structure and surface morphology of MoSe_2_ NFs. Raman spectroscopy was used to identify the composition of the MS oxidized solution. High‐performance liquid chromatography (HPLC, VARIAN 901‐MS) with a C18 inversed‐phase column was employed for detecting the content ratio of H_2_SeO_4_ and H_2_SeO_3_, and the mobile phase was prepared by ammonium acetate and methanol (3:1, v/v) mixing solution.

### Degradation Experiment

A rotary evaporator was used to prepare a high concentration H_2_O_2_ solution up to 90%. To analyze the degradation activity, the 10 mg MoSe_2_ NFs were added into the 10 mL RhB and 10 mL 90% H_2_O_2_ mixing solution. The UV–Visible spectrophotometer (Hitachi U‐3900) was then employed to measure the decomposition ratio of different dye concentration. Carbon felt (CeTech Co., Ltd., 15 × 15 cm^2^) was used as a substrate together with MoSe_2_ NFs precursor and transferred to an autoclave with PTFE lining, and maintained at 220 °C for 24 h. After that, the MoSe_2_ NFs were grown on the surface of carbon felt.

### Synthesized MoSe_2_ NFs Using the Treated Solution

After the dye was decomposed by the MS oxidized solution (as referred to as treated solution), hydrazine, which acts as the reducing agent, was added into the treated solution to adjust the pH value up to seven. The solution was transferred to an autoclave with PTFE lining and maintained at 220 °C for 24 h to obtain the MoSe_2_ NFs‐B.

### Cabbage Seedling Growth Experiment

Two germinate cabbages were irrigated in either treated or untreated solutions. The burette's tip was fixed 2 cm from the seedling to observe growth status, which was recorded entirely with a time‐lapse camera.

### Gibbs Free Energy Calculation

The Gibbs free energy change (Δ*G*) of degradation reactions was calculated by the first‐principles simulation DMol^3^ module of Materials Studio, which indicated that the degradation process was dominated by a spontaneous reaction. Generalized gradient approximation (GGA) with Perdew, Burke, and Ernzerhof exchange‐correlation functionals was employed. The Δ*G* of the reactions was calculated by the density functional theory based on the DMol^3^ module using Material Studio. First, the molecular structure of MoSe_2_, H_2_O_2_, H_2_SeO_4_, H_2_SeO_3_, RhB, MoO_3_, H_2_, O_2_, H_2_O, CO_2_, NH_3_, and Cl_2_ were built‐in from the database. Next, the GGA with BLYP (Becke‐Lee‐Yang‐Parr) exchange‐correlation functionals^[^ ^58^, ^59^ ^]^ was employed for geometry optimization and frequency analysis. After that, the total energy (total *E*), entropy (*S*), heat capacity (*C*_v_), enthalpy (*H*), and free energy (*G*) at room temperatures (298.15 K) for all reactants and products from Equations (1–, 3) were obtained by frequency analysis mode (see Table S2, Supporting Information). The Gibbs free energy changes (i.e., Δ*G*_1_, Δ*G*_2_, and Δ*G*_3_) were calculated as shown in Note S1, Supporting Information, by the following equation:

(4) $$\begin{array}{c} \Delta G^{298.15\;K}=\sum \mathrm{total}\;G^{298.15\;K}\left(\mathrm{products}\right)-\sum \mathrm{total}\;G^{298.15\;k}\left(\mathrm{reactants}\right) \end{array}$$

## Conflict of Interest

The authors declare no conflict of interest.

## Author Contributions

J.M.W. and J.C. supervised the project. J.T.L. and J.M.W. conceived the idea. J.T.L., J.M.W., and J.C. designed the experiments. J.T. conducted the experiments and theoretical calculation using Materials Studio. J.T.L., J.C., and J.M.W. analyzed the experimental data and working principles, drew the figures, and prepared the manuscript. S.M. and S.S. reviewed and made technical comments on the manuscript.

## Supporting information

Supporting Information

Supplementary VideoS1

Supplementary VideoS2

Supplementary VideoS3

## Data Availability

Data available on request from the authors
