# Supplementary material for: MoSe2 Nanoflowers for Highly Efficient Industrial Wastewater Treatment with Zero Discharge
Source: Adv Sci (Weinh). 2021 Oct 24;8(23):2102857. doi: 10.1002/advs.202102857 (PMC8655190; doi:10.1002/advs.202102857)
Supplement: Supplementary file 1 — Supporting Information [file ADVS-8-2102857-s003.pdf]

© 2021 Wiley-VCH GmbH

ADVANCED SCIENCE
Open Access

Supporting Information

for *Adv. Sci.,* DOI: 10.1002/advs.202102857

$MoSe_2$ Nanoflowers Highly Efficient Industrial Wastewater Treatment with Zero Discharge

Jyun-Ting Lee, Shaurya Mathur, Sophia Shen, Jyh Ming Wu,and Jun Chen**

Supporting Information

$MoSe_2$ Nanoflowers Highly Efficient Industrial Wastewater Treatment with Zero Discharge

Jyun-Ting Lee, Shaurya Mathur, Sophia Shen, Jyh Ming Wu, and Jun Chen**

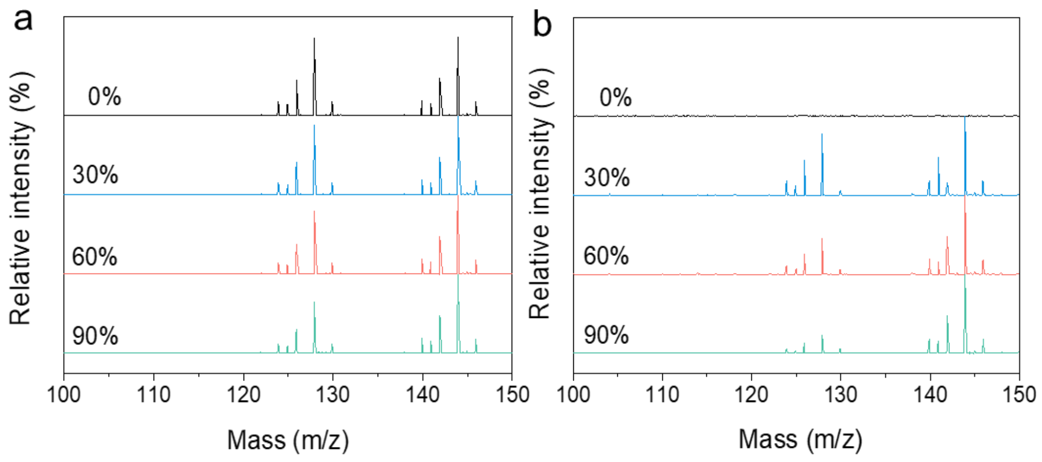

Figure S1. Concentration ratio analysis of H_2SeO_3 and H_2SeO_4. (a) The EIS-MS spectra of commercial H_2SeO_4 solution were mixed with various concentrations of H_2O_2 from 0 to 90%, respectively. Reaction condition were 10 mL commercial H_2SeO_4 solution, 10 mL H_2O_2 solution. (b) The EIS-MS spectra of $MoSe_2$ NFs were mixed with various concentrations of H_2O_2 from 0 to 90%, respectively. Reaction condition were 10 mg $MoSe_2$ NFs, 10 mL H_2O_2.

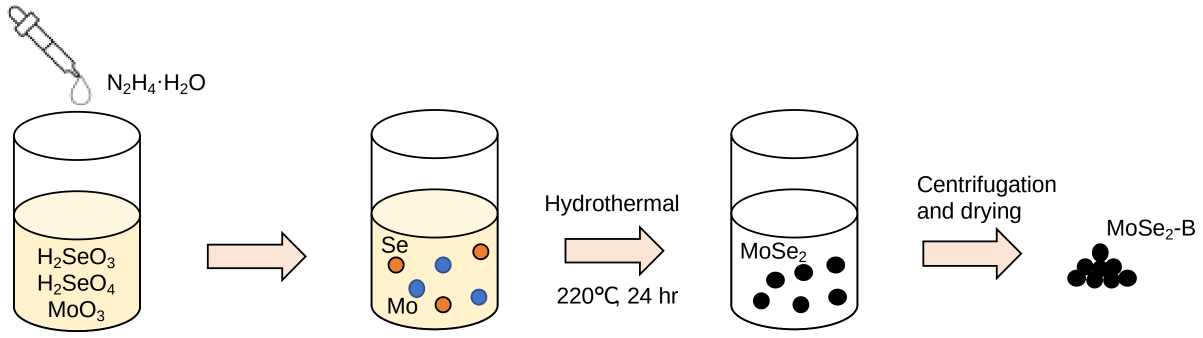

Figure S2. The process of $MoSe_2$-B reconstruction.

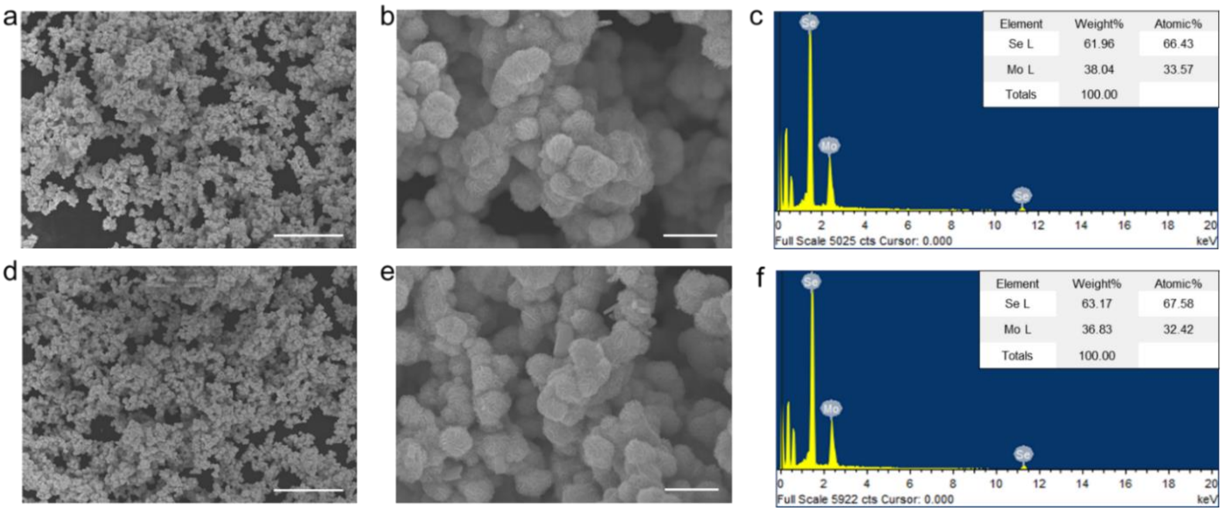

Figure S3. Surface morphology of $MoSe_2$-A and -B. (a) Low magnification, (b) high magnification SEM images, and (c) the EDS spectra of $MoSe_2$-A NFs. Scale bar: 25 μm and 2 μm, respectively. (d) Low magnification, (e) high magnification SEM images, and (f) the EDS spectra of $MoSe_2$-B NFs. Scale bar: 25 μm and 2 μm, respectively.

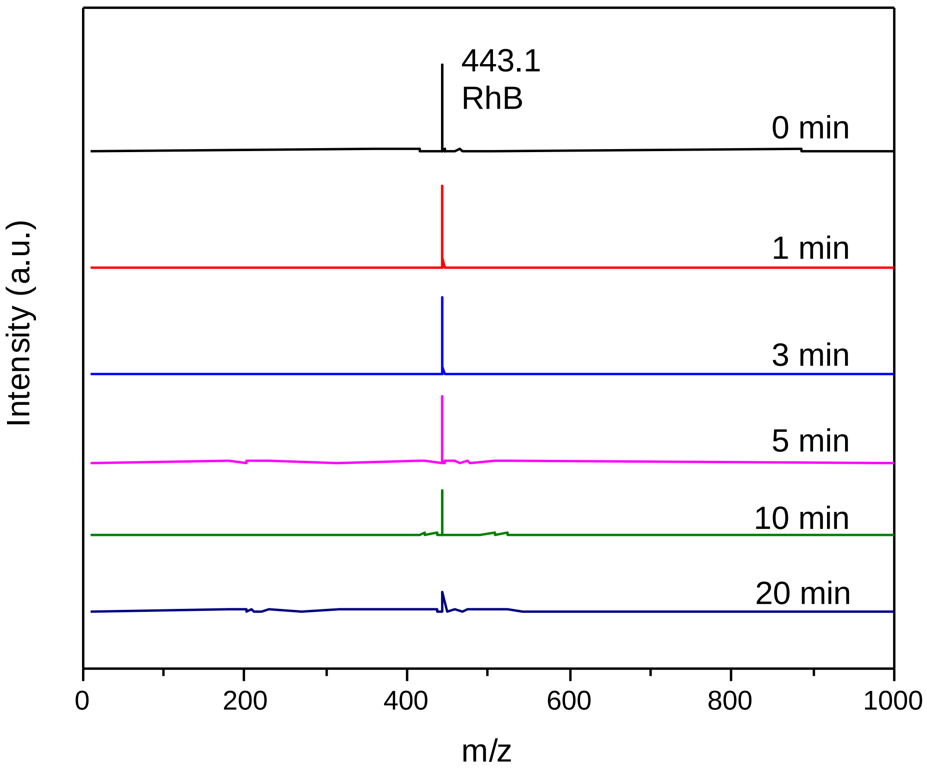

Figure S4. The Positive ion ESI mass spectra in the photooxidation of RhB intermediates in the presence of TiO_2/SiO_2 under visible light irradiation.

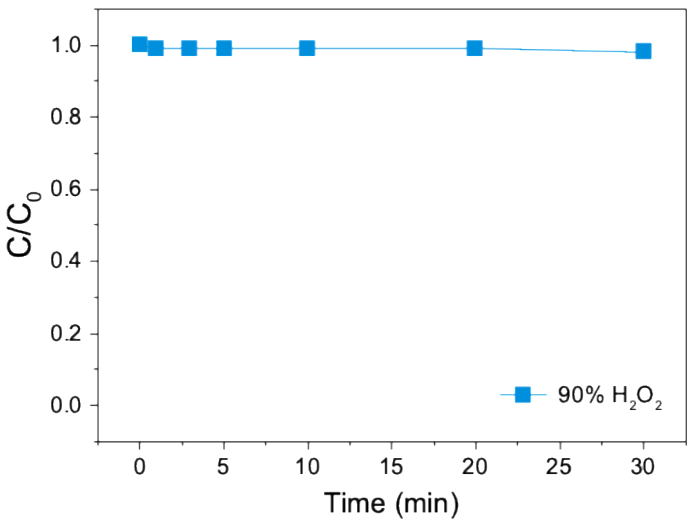

Figure S5. Degradation performance of the control group. The degradation curve of 90% H_2O_2. Reaction conditions were 10 mL 90% H_2O_2 solution, 10 mL RhB solution.

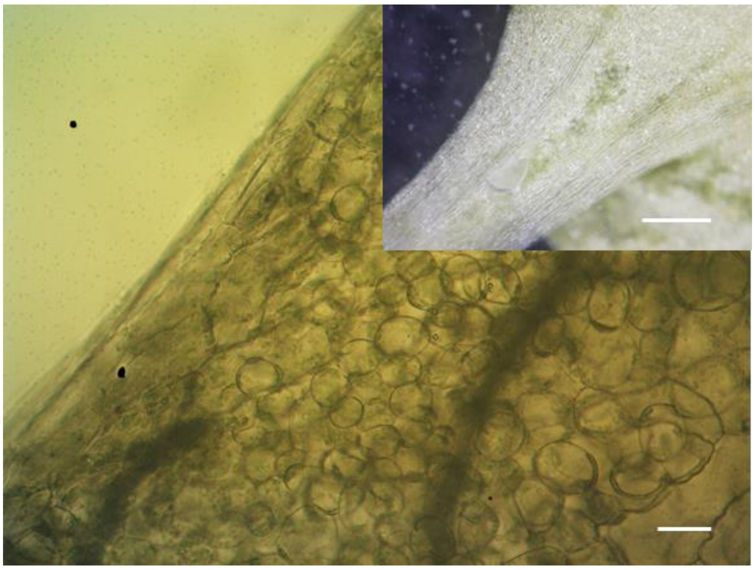

Figue S6. Control experiment of the cabbage seedlings growing. The OM image of normal leaf. Scale bar: 5 μm. Inset, the digital image of the leaf. Scale bar: 1 mm.

Table S1. The comparison of degradation performance in recent 5 years.

Material	Mechanism	Weight	Volume	Concentration	Reference
Core–shell CFO–BFO NPs	Magnetoelectrically driven catalytic	20 mg	20 mL	2 ppm	45
$ZnSnO_3$ nanowires	Piezo-photocatalytic	10 mg	10 mL	10 ppm	46
MoS_2	Piezocatalytic	10 mg	50 mL	10 ppm	34
Na-, K-, and Cs-salts of heptazine imide	Photocatalytic	5 mg	5 mL	20 ppm	47
Barbituric acid	Photocatalytic	5 mg	20 mL	20 ppm	48
Rotating triboelectric nanogenerator (R-TENG)	Electrochemical degradation	-	25 mL	100 ppm	49
Quartz Microrods@MoS_2	Piezocatalytic	10 mg	10 mL	10 ppm	50
Triboelectric nanogenerators (TENGs)	Electrochemical degradation	-	200 mL	-	51
$MoSe_2/H_2O_2$	Photocatalytic	50 mg	100 mL	20 ppm	52
$MoSe_2/H_2O_2$	Cyclic chemical reactions	10 mg	10 mL	1500 ppm	This work

Table S2. The thermodynamic properties at 298.15 K and 1 atm obtained by $Dmol^3$ calculation.

	$MoSe_2$	H_2O_2	RhB	H_2SeO_3	H_2SeO_4	MoO_3	NH_3	Cl_2	H_2	O_2	H_2O	CO_2
G (kcal/mol)	-3.043	-14.032	-35.904	-14.058	-18.28	-17.65	-11.99	-14.155	-7.624	-12.32	-11.502	-13.422
G (eV)	-0.131	-0.608	-1.556	-0.609	-0.792	-0.765	-0.519	-0.613	-0.330	-0.534	-0.498	-0.582

Supplementary Note S1

To obtain a continuously Gibbs free energy change profile, the calculation should be following the law of conservation of mass. Thus, the Eq. (1)-(3) were rewritten as following 4 steps.

Step 1:

$$0.5MoSe_2 + 3.75H_2O_2 + \frac{2}{131}C_{28}H_{31}ClN_2O_3$$

$$\downarrow$$

$$2MoSe_2 + 11H_2O_2 \xrightarrow{\Delta G_1} 4H_2SeO_3 + 2MoO_3 + 7H_2 + 2O_2 \quad (1)$$

Step 2:

$$H_2SeO_3 + 0.5MoO_3 + 1.75H_2 + 0.5O_2 + \frac{2}{131}C_{28}H_{31}ClN_2O_3 + H_2O_2$$

$$H_2SeO_3 + H_2O_2 \xrightarrow{\Delta G_2} H_2SeO_4 + H_2O \quad (2)$$

Step 3:

$$0.5MoO_3 + 1.75H_2 + 0.5O_2 + \frac{2}{131}C_{28}H_{31}ClN_2O_3 + H_2SeO_4 + H_2O$$

$$131H_2SeO_4 + 2C_{28}H_{31}ClN_2O_3 \xrightarrow{\Delta G_3} 131H_2SeO_3 + 56CO_2 + 4NH_3 + Cl_2 + 25H_2O \quad (3)$$

Step 4:

$$0.5MoO_3 + 1.75H_2 + 0.5O_2 + H_2SeO_3 + \frac{56}{131}CO_2 + \frac{4}{131}NH_3 + \frac{1}{131}Cl_2 + \frac{156}{131}H_2O$$

The G of step 1 was assumed as 0, and ΔG_1, ΔG_2, and ΔG_3 were calculated from step 1 to 2, step 2 to 3, and step 3 to 4, respectively.
